# Uptake of the NICE osteoarthritis guidelines in primary care: a survey of older adults with joint pain

**DOI:** 10.1186/s12891-018-2196-2

**Published:** 2018-08-17

**Authors:** Emma Louise Healey, Ebenezer K. Afolabi, Martyn Lewis, John J. Edwards, Kelvin P. Jordan, Andrew Finney, Clare Jinks, Elaine M. Hay, Krysia S. Dziedzic

**Affiliations:** 1 Research Institute for Primary Care and Health Sciences, Keele University, Keele, Staffordshire ST5 5BG UK; 2 Keele Clinical Trials Unit, David Weatherall Building, Keele University, Staffordshire, UK; 3 School of Nursing and Midwifery, Keele University, Staffordshire, UK

**Keywords:** Osteoarthritis, Joint pain, General practice, NICE guidelines

## Abstract

**Background:**

Osteoarthritis (OA) is a leading cause of pain and disability. NICE OA guidelines (2008) recommend that patients with OA should be offered core treatments in primary care. Assessments of OA management have identified a need to improve primary care of people with OA, as recorded use of interventions concordant with the NICE guidelines is suboptimal in primary care. The aim of this study was to i) describe the patient-reported uptake of non-pharmacological and pharmacological treatments recommended in the NICE OA guidelines in older adults with a self-reported consultation for joint pain and ii) determine whether patient characteristics or OA diagnosis impact uptake.

**Methods:**

A cross-sectional survey mailed to adults aged ≥45 years (*n* = 28,443) from eight general practices in the UK as part of the MOSAICS study. Respondents who reported the presence of joint pain, a consultation in the previous 12 months for joint pain, and gave consent to medical record review formed the sample for this study.

**Results:**

Four thousand fifty-nine respondents were included in the analysis (mean age 65.6 years (SD 11.2), 2300 (56.7%) females). 502 (12.4%) received an OA diagnosis in the previous 12 months. More participants reported using pharmacological treatments (e.g. paracetamol (31.3%), opioids (40.4%)) than non-pharmacological treatments (e.g. exercise (3.8%)). Those with an OA diagnosis were more likely to use written information (OR 1.57; 95% CI 1.26,1.96), paracetamol (OR 1.30; 95% CI 1.05,1.62) and topical NSAIDs (OR 1.30; 95% CI 1.04,1.62) than those with a joint pain code. People aged ≥75 years were less likely to use written information (OR 0.56; 95% CI 0.40,0.79) and exercise (OR 0.37; 95% CI 0.25,0.55) and more likely to use paracetamol (OR 1.91; 95% CI 1.38,2.65) than those aged < 75 years.

**Conclusion:**

The cross-sectional population survey was conducted to examine the uptake of the treatments that are recommended in the NICE OA guidelines in older adults with a self-reported consultation for joint pain and to determine whether patient characteristics or OA diagnosis impact uptake. Non-pharmacological treatment was suboptimal compared to pharmacological treatment. Implementation of NICE guidelines needs to examine why non-pharmacological treatments, such as exercise, remain under-used especially among older people.

## Background

Osteoarthritis (OA) is a leading cause of pain and morbidity and, globally, is the fastest increasing cause of disability [1]. Evidence is accumulating about how primary care could reduce OA pain and disability: international guidelines address best evidence for components of this care but their impact on practice and behaviour is not clearly understood. In order to investigate this, we assessed uptake of one national (United Kingdom (UK)) OA management guideline by the general population.

The UK National Institute for Health and Care Excellence (NICE) produced OA management recommendations in 2008 [2], focused on the peripheral joint sites of the hip, knee, hand and foot. The NICE working definition of OA (here, “clinical OA”) is based upon symptoms of activity-related joint pain rather than radiographic signs. The majority of self-reported joint pain in older adults has been determined to be due to clinical OA, with alternative clear diagnoses being relatively uncommon [3].

One-in-ten older people will consult primary care every year for clinical OA (diagnosed OA or recorded peripheral joint pain) [4]. NICE OA guidelines suggest that all core treatments (education, exercise, and weight loss) should be offered to everyone, irrespective of age, pain severity and co-morbidity [2]. Assessments of OA management have identified a need to improve primary care of people with OA, as recorded use of interventions concordant with the NICE guidelines is suboptimal [5]. The challenge for primary care is how best to manage OA for the majority of people [6, 7].

The aims of this study were, without prior hypothesis, i) to describe the patient-reported uptake of non-pharmacological and pharmacological treatments recommended in the NICE OA guidelines in a community-dwelling older adult population with a self-reported primary care consultation for joint pain, and, ii) to determine whether patient characteristics (age, sex, self-reported health, number of sites of disease, and overall morbidity burden) or a recorded formal diagnosis of OA were associated with uptake of these treatments.

## Methods

### Study design and population

This paper is one component of the ‘Management of OSteoArthritis In ConsultationS’ (MOSAICS) study [8, 9]. The data were derived from a cross-sectional population survey. A linked medical record review was conducted in order to estimate morbidity burden and identify the presence of any formal OA diagnosis. The findings are reported in line with the STROBE guidelines [10].

A 12 month period for diagnosis and consultation was selected to maximise accurate recall, to reflect those who had recently sought health care, and to include experience since the NICE guidelines had been published.

### Data collection

#### The population survey

The population survey was mailed between May 2011 and April 2012 to all adults aged ≥45 years (*n* = 28,443) registered with eight general practices in the West Midlands and North West of England that varied in the size of the registered population, clinical staffing, urbanization and deprivation.

The survey used a two-stage mailing process. Prior to the mailing, General Practitioners (GPs) screened the list of potential participants and excluded people considered not eligible (e.g. those with psychiatric illness, recent bereavement). A letter of invitation to participate, study information, and the survey were sent to all eligible people. Individuals were invited to complete the survey and return it in a pre-paid envelope, indicating whether they consented to further contact and medical record review. A reminder letter and additional copy of the survey were sent to non-responders after 3 weeks. A telephone contact number provided recipients the opportunity to place questions and opt-out if they wished.

Survey responders provided socio-demographic and general health information and were asked to indicate whether they had experienced joint pain (hip, knee, hand and foot) in the previous 12 months. Those confirming the presence of joint pain were asked to report their consultation behaviours and treatment(s) used for their joint pain over the previous 12 months. Everyone who both reported a consultation for joint pain and consented to medical record review formed the study population for this analysis.

Survey responders were asked their gender, date-of-birth (for calculation of current age), height and weight, whether they lived alone, and work status (employed, unemployed, retired). General health was assessed using the SF-12 [11], a validated, generic measure with two summary scales: the physical component summary (PCS) and the mental component summary (MCS), standardised to scores from the general population of the United States (mean = 50, where lower scores indicate worse health).

Presence of joint pain over the previous 12 months was based on single questions for each of the peripheral joint sites of interest (hip, knee, hand and foot). For example, participants were asked, “Have you had any pain in the last year in or around the hip? (Yes/No)” (modified from Jinks et al., 2004) [12]. Those reporting pain in two or more of the four sites were classed as having multi-site joint pain.

Participants were asked if they had consulted their GP or practice nurse (PN) regarding joint pain over the previous 12 months. Self-reported information regarding the management of their joint pain over the previous 12 months was also collected. Participants were asked “In the past 12 months have you tried any of these for your joints?” Patients were asked to tick boxes to indicate which treatments had been used (modified from Jinks et al., 2004) [12]. Following this question was a list of options which linked to the NICE guidance e.g. joint operation, use of treatments such as core non-pharmacological treatments, and first- and second-line pharmacological treatments (see Table 2).

#### Medical record review

A retrospective medical record review in the study population was conducted to identify all Read codes recorded in consultations in the previous 12 months. Read codes are the most common way of recording morbidity in UK primary care. Anyone with a Read code from the N05 “Osteoarthritis and allied disorders” branch recorded in that period was classed as having a formal OA diagnosis.

To determine the morbidity burden, polypharmacy was employed as a simple proxy measure [13]. The count of unique drug types from the British National Formulary (BNF) prescribed in the previous 12 months was obtained from the medical record. Patients were dichotomised into two groups: < 10 and ≥ 10  drug types, based on previous work [14].

### Statistical analysis

The study population was described in terms of socio-demographic factors, health status, recorded formal OA diagnosis in the last 12 months, morbidity burden, and number of self-reported joint pain sites (dichotomised into single or multiple). Uptake of recommended treatments was described in the study population, stratified by responder age-group.

Descriptive statistics were used, with mean and standard deviation (SD), frequency counts and percentages (as appropriate) presented. Age was grouped by decades and skewed data such as the SF12-PCS and SF12-MCS were categorised based on quartile scores to determine any association between health status and uptake of recommended treatments. A chi-squared test-for-trend was employed to estimate relationships between uptake of recommended treatments and age group.

Multivariable logistic regression analyses were carried out to estimate associations between participants’ socio-demographic and health factors and the uptake of non-pharmacological and pharmacological treatments. The multivariate model was fully inclusive of all variables listed in Table 3. Results are shown as fully-adjusted odds ratios (AOR) with 95% confidence intervals (CI).

Data analysis was performed using IBM SPSS Statistics version 21 (Armonk, NY, USA) and STATA version 13 (StataCorp, 2013).

## Results

**Fig. 1:**
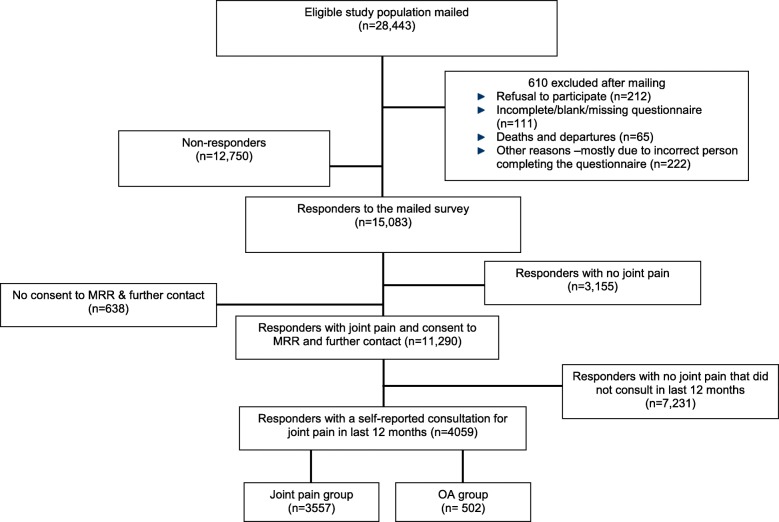
Flow chart of MOSAICS population survey

Of the 28,443 people mailed the survey, 15,083 (53%) responded. Non-responders were more likely to be male (difference in response, 10.4%) and younger (mean difference 5.1 years). There were 11,290 participants with relevant self-reported joint pain who consented to medical record review (75%). 4059 (36%) reported consulting primary care for their joint pain in the previous 12 months and these formed the study population for this paper (Fig. 1). 502 (12.4%) were found to have a formal OA diagnosis in their medical record in the previous 12 months. Table 1 shows the characteristics of participants included in the study population.

**Table 1:** Characteristics of the eligible population

Characteristic	Participants (*n* = 4059)
Gender	
Female	2300 (56.7)
Male	1759 (43.3)
Age (years)	
45–54	770 (19.0)
55–64	1136 (28.0)
65–74	1225 (30.2)
75 and above	928 (22.8)
^**a**^Employment status	
Employed	1105 (27.9)
Unemployed	596 (15.1)
Retired	2253 (57.0)
^**a**^BMI (kg/m^2^)	
Not overweight (< 25.0)	1161 (29.6)
Overweight (25.0–29.9)	1583 (40.3)
Obese (≥30.0)	1184 (30.1)
No. of pain sites	
Single site	777 (19.1)
Multi-site	3282 (80.9)
Morbidity burden (BNF drug count)	
< 10 count	2222 (54.7)
≥ 10 count	1837 (45.3)
SF12 - Physical health, mean (SD)	43.6 (12.4)
SF12 - Mental health, mean (SD)	49.5 (10.5)
OA diagnosis	
Yes	502 (12.4)
No	3557 (87.6)

^a^Distribution based on valid response (missing data: 105, 2.6% (employment status); 131, 3.2% (BMI); 107, 2.6% (SF-12))

Table 2 describes the uptake of all of the NICE-recommended treatments in the previous 12 months. Overall the uptake of the core non-pharmacological treatments was considerably lower than the first-line pharmacological treatments. For example, only 9.4 and 3.8% of patients used weight loss or aerobic fitness training respectively, whereas 31.3 and 26.0% of patients used paracetamol or topical non-steroidal anti-inflammatory drugs (NSAIDs) respectively.

**Table 2:** Uptake of the NICE recommended treatments in the past 12 months, overall and stratified by age groups

	Total (*n* = 4059)		45-54y n (%)	55-64y n (%)	65-74y n (%)	≥75y n (%)
	n	% (95% CI)				
Core treatments						
^***^Written information^a^	934	23.0 (21.7,24.3)	190 (24.7)	298 (26.2)	267 (21.8)	179 (19.3)
^**^Muscle strengthening exercises	532	13.1 (12.1,14.1)	113 (14.7)	172 (15.1)	150 (12.2)	97 (10.5)
^*^Aerobic fitness exercise	154	3.8 (3.2,4.4)	36 (4.7)	44 (3.9)	55 (4.5)	19 (2.0)
Dieting to lose weight^b^	261	9.4 (8.3,10.5)	37 (7.1)	90 (10.9)	99 (11.4)	35 (6.4)
1st and 2nd line pharmacological treatment						
^***^Paracetamol	1270	31.3 (29.9,32.7)	149 (19.4)	283 (24.9)	395 (32.2)	443 (47.7)
^***^Anti-inflammatory creams/gels e.g. topical NSAIDs	1055	26.0 (24.7,27.4)	150 (19.5)	243 (21.4)	341 (27.8)	321 (34.6)
^*^Capsaicin cream	66	1.6 (1.2,2.0)	4 (0.5)	22 (1.9)	21 (1.7)	19 (2.0)
^***^Anti-inflammatory tablets e.g. oral NSAIDs	1276	31.4 (30.0,32.8)	306 (39.7)	406 (35.7)	366 (29.9)	198 (21.3)
^***^Stronger painkillers e.g. opioids, compound analgesics	1641	40.4 (38.9,41.9)	263 (34.2)	416 (36.6)	535 (43.7)	427 (46.0)
Intra-articular corticosteroid injection	463	11.4 (10.4,12.4)	81 (10.5)	146 (12.9)	131 (10.7)	105 (11.3)
Adjunctive treatment						
Warmth, heat or cold application	334	8.2 (7.4,9.0)	57 (7.4)	105 (9.2)	95 (7.8)	77 (8.3)
^***^Walking aids	676	16.7 (15.6,17.8)	64 (8.3)	122 (10.7)	179 (14.6)	311 (33.5)
^***^Assistive devices	301	7.4 (6.6,8.2)	24 (3.1)	44 (3.9)	75 (6.1)	158 (17.0)
Transcutaneous electric nerve stimulation (TENS)	127	3.1 (2.6,3.6)	22 (2.9)	41 (3.6)	37 (3.0)	27 (2.9)
Shock-absorbing shoes or insoles	247	6.1 (5.4,6.8)	41 (5.3)	63 (5.5)	81 (6.6)	62 (6.7)
Appliances and support and braces	265	6.5 (5.7,7.3)	52 (6.8)	80 (7.0)	59 (4.8)	74 (8.0)
Service use						
Joint arthroplasty/operation	365	9.0 (8.1,9.9)	73 (9.5)	105 (9.2)	102 (8.3)	85 (9.2)
^***^Manual therapy	998	24.6 (23.3,25.9)	219 (28.4)	311 (27.4)	282 (23.0)	186 (20.0)

^a^Written information is a composite variable derived from the addition of responses of participants who used information about treatments, information about self-management and information about OA when they consulted with joint pain

^b^Restricted to obese/overweight participants (*n* = 2767)

Treatment association with age is indicated by: ^*^p < 0.05, ^**^p < 0.01, ^***^p < 0.001 (by chi square test for trend)

The multivariable analysis demonstrated that various patient characteristics were associated with uptake of recommended treatments (see Table 3): women, compared to men, were more likely to report use of written information (AOR 1.28, 95% CI 1.09,1.50) and weight loss (AOR 1.54, 95% CI 1.16,2.04). Older individuals (≥75 compared to age 45–54) were less likely to report use of written information (AOR 0.56, 95% CI 0.40,0.79) and exercise (AOR 0.37, 95% CI 0.25,0.55), but more likely to report use of paracetamol (AOR 1.91, 95% CI 1.38,2.65). Multi-site joint pain was associated with greater provision of information only. Those with a greater morbidity burden (≥10 BNF count of unique drug types compared to < 10) were more likely to use exercise (AOR 1.44, 95% CI 1.16,1.77) and weight management (AOR 1.87, 95% CI 1.37,2.57) and both first-line pharmacological treatments. Worse scores on the SF-12 PCS (below lower quartile score compared to above upper quartile score) were associated with greater use of information (AOR 2.13, 95% CI 1.63,2.77), exercise (AOR 1.64, 95% CI 1.20,2.24), and both first-line pharmacological treatments. A similar comparison for the SF-12 MCS suggested greater use of information, weight loss and both pharmacological treatments in people with worse scores. Those with a recorded diagnosis of OA were more likely to report use of information and both first-line pharmacological treatments.

**Table 3:** Uptake of the recommended NICE core non-pharmacological and first line pharmacological treatments (*N* = 4059)

Characteristic	^a^Written information		Muscle strengthening/ aerobic fitness exercise		^b^Dieting to lose weight		Paracetamol		Topical NSAIDs	
	n (%)	AOR (95% CI)	n (%)	AOR (95% CI)	n (%)	AOR (95% CI)	n (%)	AOR (95% CI)	n (%)	AOR (95% CI)
Total	934		618		261		1270		1055	
Gender										
Male	353 (20.1)	1	244 (13.9)	1	92 (7.1)	1	511 (29.1)	1	424 (24.1)	1
Female	581 (25.3)	1.28^××^ (1.09, 1.50)	374 (16.3)	1.12 (0.93, 1.35)	169 (11.5)	1.54^××^ (1.16, 2.04)	759 (33.0)	1.09 (0.94, 1.27)	631 (27.4)	1.13 (0.97, 1.32)
Age group (years)										
45–54	190 (24.7)	1	133 (17.3)	1	37 (7.1)	1	149 (19.4)	1	150 (19.5)	1
55–64	298 (26.2)	1.02 (0.80, 1.29)	192 (16.9)	0.78 (0.60, 1.03)	90 (10.9)	1.52 (0.97, 2.36)	283 (25.0)	1.12 (0.87, 1.45)	243 (21.4)	0.96 (0.74, 1.24)
65–74	267 (21.8)	0.76 (0.56, 1.03)	183 (14.9)	0.55^××^ (0.38, 0.78)	99 (11.4)	1.64 (0.94, 2.86)	395 (32.2)	1.29 (0.94, 1.76)	341 (27.8)	1.20 (0.88, 1.64)
≥ 75	179 (19.3)	0.56^××^ (0.40, 0.79)	110 (11.9)	0.37^×××^ (0.25, 0.55)	35 (6.4)	0.74 (0.39, 1.40)	443 (47.7)	1.91^×××^ (1.38, 2.65)	321 (34.6)	1.32 (0.94, 1.84)
Employment status										
Employed	277 (25.1)	1	171 (15.5)	1	56 (7.1)	1	203 (18.4)	1	197 (17.8)	1
Unemployed	143 (24.0)	0.74^×^ (0.58, 0.98)	102 (17.1)	1.03 (0.77, 1.38)	49 (12.8)	1.06 (0.67, 1.66)	195 (32.7)	1.16 (0.90, 1.51)	157 (26.3)	1.17 (0.90, 1.52)
Retired	494 (21.9)	0.91 (0.71, 1.18)	327 (14.5)	1.17 (0.87, 1.58)	149 (9.7)	0.92 (0.58, 1.45)	841 (37.3)	1.15 (0.89, 1.50)	667 (29.6)	1.12 (0.86, 1.46)
No. of pain sites										
Single site	116 (14.9)	1	106 (13.6)	1	31 (6.4)	1	180 (23.2)	1	151 (19.4)	1
Multi-site	818 (24.9)	1.59^×××^ (1.26, 2.00)	512 (15.6)	0.98 (0.77, 1.26)	230 (10.1)	1.08 (0.70, 1.68)	1090 (33.2)	1.10 (0.88, 1.35)	904 (27.5)	1.15 (0.93, 1.43)
Morbidity burden (BNF drug count)										
< 10 counts	501 (22.5)	1	294 (13.2)	1	87 (5.9)	1	451 (20.3)	1	419 (18.9)	1
≥ 10 counts	433 (23.6)	0.91 (0.75, 1.08)	324 (17.6)	1.44^××^ (1.16, 1.77)	174 (13.5)	1.87^×××^ (1.37, 2.57)	819 (44.6)	1.88^×××^(1.60, 2.23)	636 (34.6)	1.61^×××^ (1.35, 1.91)
BMI (Kg/m^2^)										
Not overweight (< 25.0)	253 (21.8)	1	170 (14.6)	1	n/a	n/a	343 (29.5)	1	287 (24.7)	1
Overweight (25.0–29.9)	355 (22.4)	1.01 (0.83, 1.23)	246 (15.5)	1.02 (0.82, 1.27)	84 (5.3)	1	482 (30.4)	1.05 (0.87, 1.27)	420 (26.5)	1.12 (0.93, 1.35)
Obese (≥30.0)	295 (24.9)	0.96 (0.78, 1.18)	180 (15.2)	0.80 (0.63, 1.02)	177 (15.0)	2.48^×××^ (1.85, 3.32)	388 (32.8)	0.95 (0.78, 1.16)	311 (26.3)	0.91 (0.74, 1.12)
SF12 – Physical health (Quartile)										
Worst to 27.58	273 (27.7)	2.13^×××^ (1.63, 2.77)	168 (17.0)	1.64^××^ (1.20, 2.24)	102 (14.2)	1.44 (0.89, 2.32)	467 (47.4)	2.64^×××^ (2.04, 3.41)	344 (34.9)	1.67 × × × (1.28, 2.16)
27.59–38.36	249 (25.4)	1.76^×××^ (1.38, 2.25)	167 (17.0)	1.67 ^×××^(1.26, 2.23)	75 (11.0)	1.29 (0.81, 2.05)	361 (36.8)	2.24^×××^ (1.75, 2.87)	290 (29.6)	1.60 × × × (1.25, 2.04)
38.37–48.26	231 (23.1)	1.53^×××^ (1.21, 1.93)	155 (15.5)	1.47^××^ (1.12, 1.93)	42 (6.2)	0.86 (0.53, 1.40)	261 (26.1)	1.70^×××^ (1.33, 2.16)	225 (22.5)	1.26 (0.99, 1.61)
48.27–71.88	166 (16.9)	1	115 (11.7)	1	34 (5.5)	1	139 (14.1)	1	156 (15.8)	1
SF12 – Mental health (Quartile)										
Worst to 39.93	275 (28.5)	1.42^××^ (1.12, 1.78)	176 (18.2)	1.11 (0.85, 1.44)	100 (14.0)	1.90^××^ (1.26, 2.84)	404 (41.9)	1.59^×××^ (1.28, 1.97)	314 (32.5)	1.51^×××^ (1.21, 1.89)
39.94–49.55	231 (23.5)	1.18 (0.95, 1.48)	140 (14.3)	0.97 (0.75, 1.26)	68 (10.6)	1.64^×^(1.08, 2.48)	337 (34.3)	1.34^××^ (1.08, 1.65)	290 (29.5)	1.48^×××^ (1.18, 1.84)
49.56–57.11	207 (21.4)	1.14 (0.91, 1.43)	144 (14.9)	1.04 (0.80, 1.35)	43 (6.9)	1.10 (0.70, 1.74)	233 (24.1)	1.04 (0.84, 1.30)	203 (21.0)	1.09 (0.87, 1.37)
57.12 to 74.46	206 (19.8)	1	145 (14.0)	1	42 (5.8)	1	254 (24.5)	1	208 (20.0)	1
OA diagnosis										
Yes	146 (29.1)	1.57^×××^ (1.26, 1.96)	88 (17.5)	1.31^×^ (1.00, 1.70)	31 (8.9)	0.79 (0.52, 1.21)	203 (40.4)	1.30^×^ (1.05, 1.62)	166 (33.1)	1.30^×^ (1.04, 1.62)
No	788 (22.2)	1	530 (14.9)	1	230 (9.5)	1	1067 (30.0)	1	889 (25.0)	1

n is the number of participants who used core treatments out of 4059 eligible respondents. Number in the subcategories do not always sum to the total number of who used treatment due to missing data. % figures represent valid percent (i.e. excluding missing data)

*AOR* adjusted odds ratio

^a^Written information is a composite variable derived from the addition of responses of participants who used information about treatments, information about self-management and information about OA when they consulted with joint pain; ^b^Restricted to obese/overweight participants. *n* = 3742 in final multivariable analysis

^×^*p* < 0.05, ^××^*p* < 0.01, ^×××^*p* < 0.001

## Discussion

### Summary

This study examined one example of a national guideline of best primary care for OA and found evidence that those with OA report a lack of guideline-based advice and treatment; a finding that was particularly predominant in the oldest ages. This is similar to treatment patterns for knee pain demonstrated prior to 2008 [15]. Semi-structured interviews with older adults with knee pain in a 2008 study had identified an early reliance on pharmacological treatments and underuse of non-pharmacological interventions in early treatment choices [15]. Exercise of any type for OA has also previously been found to be under-used in primary care [16].

### Strengths and limitations

A strength of this survey is the large sample size achieved ensuring greater precision in estimates and sufficient power to test statistical associations. Use of self-reported information has some advantages to medical record use as non-pharmacological interventions and over-the-counter drug use are poorly-recorded in medical records. The heterogeneity of practice characteristics across the sample increases the generalisability of the findings to the UK population as a whole. Due to the nature of the data collection, one potential limitation of this study is recall bias. A recall period of up to 12 months may have affected participants’ ability to accurately self-report information about their consultation behaviours and treatments used. As the study focused on treatments over the previous 12 months, it was also impossible to determine whether other treatments had been tried prior to moving onto further treatments (e.g. a trial of non-pharmacological treatments prior to first-line pharmacological options). It is not known whether people reporting use of treatments were responding to clinical recommendations or acting independently; for those not using treatments, they may not have been advised to do so or chosen not to. Although we performed multiple comparisons, the main conclusions rest on plausible and consistent associations across ages and comparable aspects of care. The NICE 2008 guidelines have been updated in 2014 [17]. Although the patient survey data were collected before the 2014 update, there is no reason to suspect that clinical practice would be particularly different since the guideline update, especially since the emphasis on non-pharmacological strategies is retained in the 2014 update. The issues raised by the findings of this survey remain very relevant.

### Comparison with existing literature

This survey considered everyone with a self-reported consultation for joint pain in the previous 12 months. Only 12.4% of the study population had a recorded OA diagnosis in their medical record during this period, though study participants may have received an OA diagnosis prior to this. People with recorded peripheral joint pain have previously been identified to have a similar preponderance of radiographic OA compared to those with an OA diagnosis [18] and so it is reasonable to consider that the OA guidelines would apply to the whole study population.

In the adjusted models, which accounted inter alia for sex, age, morbidity burden, and clinical severity (through the SF-12 PCS and multi-site pain variable, as multi-site pain is associated with symptom severity [19]), the main variable associated with lower likelihood of use of the core non-pharmacological treatments was older age. The association between older age and reduced use of information may reflect a duration effect, if older people had used it previously, though it could also be due to other factors.

It is possible that the lower use of exercise was due to patient or clinician beliefs about the appropriateness of exercise in the elderly. It has previously been identified that only 16% of men and 12% of women aged ≥65 in the general population achieve recommended physical activity levels [20], therefore while this finding is not surprising, it is in contrast to the NICE universal recommendation for exercise in people with OA despite age.

Increasing age was also associated with greater use of paracetamol. Strauss et al. [21] demonstrated that patients with a preference towards the pharmacological options were generally older, though in this study it is not known if greater paracetamol use is influenced by patient- or clinician-level management. Clinical severity, measured by the SF-12 PCS and the multisite pain variable, appears to be associated with greater use of information. Worse physical function was also associated with greater use of exercise and first-line pharmacotherapy. The latter finding is unsurprising but it is encouraging that people with worse physical function reported greater use of exercise.

### Implications for research and/or practice

This study indicates the potential importance of an OA diagnosis. People with such a diagnosis recently recorded were more likely to report having used treatments recommended in the NICE OA guidelines, i.e. certain core non-pharmacological treatments (exercise and provision of written information) and the first-line pharmacological treatments. This corresponds with previous work by our group which showed that those with an OA diagnosis were more likely to have clinician-recorded quality indicators of care than those with a joint pain symptom code [22]. It raises the possibility that, when GPs themselves are clearer about the diagnosis, there may be better uptake of the treatments that are recommended in the NICE OA guidelines by the patient, which reflects other findings about the nature of OA consultations in primary care [23].

Clinically, the known benefits of exercise [24] and weight loss [25] for hip and knee OA need to be better integrated into routine clinical practice to help reduce the apparent suboptimal uptake in the population with joint pain at large, and in the elderly in particular. Patients and clinicians need to be aware of the benefits of non-pharmacological interventions, to access these early in the course of OA and avoid reliance on pharmacological management.

A particular challenge will be to determine how to maximise patient participation in and adherence to exercise in the long-term. Evidence of barriers and facilitators of exercise adherence related to OA is not strong, although systematic reviews have highlighted the importance of educational and behavioural strategies as well as regular individualised exercise, supervision and follow-up [26, 27]. Future interventions incorporating these components should be tested to find the best way of increasing and maintaining exercise levels in this population in the long-term.

## Conclusions

This is the first survey conducted to identify self-reported use of non-pharmacological and pharmacological treatments recommended in the NICE OA guidelines within primary care. Non-pharmacological treatment uptake was found to be suboptimal and lower than pharmacological treatment, especially in older people. Effective strategies to promote guideline adherence in all ages need to be identified, with a particular emphasis on non-pharmacological management in older age groups.

## Abbreviations

AOR: Adjusted Odds Ratio
BNF: British National Formulary
CI: Confidence Interval
GP: General Practitioner
MCS: Mental Component Summary
MOSAICS: Management of OSteoArthritis In ConsultationS study
NICE: National Institute for Health & Care Excellence
NSAIDs: Non-steroidal anti-inflammatory drugs
OA: Osteoarthritis
PCS: Physical Component Summary
PN: Practice Nurse
SD: Standard Deviation
UK: United Kingdom
